# Are the Risk of Generalizability Biases Generalizable? A Meta-Epidemiological Study

**DOI:** 10.21203/rs.3.rs-3897976/v1

**Published:** 2024-02-26

**Authors:** Lauren von Klinggraeff, Chris D. Pfledderer, Sarah Burkart, Kaitlyn Ramey, Michal Smith, Alexander C. McLain, Bridget Armstrong, R. Glenn Weaver, Anthony Okely, David Lubans, John P.A. Ioannidis, Russell Jago, Gabrielle Turner-McGrievy, James Thrasher, Xiaoming Li, Michael W. Beets

**Affiliations:** Augusta University, Augusta University; University of Texas Health Science Center at Houston; University of South Carolina; University of South Carolina; University of South Carolina; University of South Carolina; University of South Carolina; University of South Carolina; University of Wollongong; University of Jyväskylä; Stanford University, Meta-Research Innovation Center at Stanford (METRICS); University of Bristol; University of South Carolina; University of South Carolina; University of South Carolina; University of South Carolina

**Keywords:** preliminary studies, bias, intervention, scaling

## Abstract

**Background:**

Preliminary studies (e.g., pilot/feasibility studies) can result in misleading evidence that an intervention is ready to be evaluated in a large-scale trial when it is not. Risk of Generalizability Biases (RGBs, a set of external validity biases) represent study features that influence estimates of effectiveness, often inflating estimates in preliminary studies which are not replicated in larger-scale trials. While RGBs have been empirically established in interventions targeting obesity, the extent to which RGBs generalize to other health areas is unknown. Understanding the relevance of RGBs across health behavior intervention research can inform organized efforts to reduce their prevalence.

**Purpose:**

The purpose of our study was to examine whether RGBs generalize outside of obesity-related interventions.

**Methods:**

A systematic review identified health behavior interventions across four behaviors unrelated to obesity that follow a similar intervention development framework of preliminary studies informing larger-scale trials (i.e., tobacco use disorder, alcohol use disorder, interpersonal violence, and behaviors related to increased sexually transmitted infections). To be included, published interventions had to be tested in a preliminary study followed by testing in a larger trial (the two studies thus comprising a study pair). We extracted health-related outcomes and coded the presence/absence of RGBs. We used meta-regression models to estimate the impact of RGBs on the change in standardized mean difference (ΔSMD) between the preliminary study and larger trial.

**Results:**

We identified sixty-nine study pairs, of which forty-seven were eligible for inclusion in the analysis (k = 156 effects), with RGBs identified for each behavior. For pairs where the RGB was present in the preliminary study but removed in the larger trial the treatment effect decreased by an average of ΔSMD=−0.38 (range − 0.69 to −0.21). This provides evidence of larger drop in effectiveness for studies containing RGBs relative to study pairs with no RGBs present (treatment effect decreased by an average of ΔSMD =−0.24, range − 0.19 to −0.27).

**Conclusion:**

RGBs may be associated with higher effect estimates across diverse areas of health intervention research. These findings suggest commonalities shared across health behavior intervention fields may facilitate introduction of RGBs within preliminary studies, rather than RGBs being isolated to a single health behavior field.

## Background

Biobehavioral research has had relatively limited impact on population health, despite large promises.(1, 2) This may be due, in part, to an observable pattern wherein behavior change interventions with promising effects in early, often small, preliminary studies show reduced or no effects when evaluated with a larger number of participants.(3, 4) Meta-studies of behavior interventions indicate external validity biases, here called Risk of Generalizability Biases (RGBs), may contribute to these reduced effects.(5, 6) RGBs are study features introduced in a preliminary study that beneficially influence observed outcomes from the preliminary study and are unlikely to be included in a larger-scale evaluation, thereby diminishing the effects observed in the larger-trial. A hallmark example of an RGB is the delivery of an intervention by a highly trained expert during a preliminary study, followed by a larger-scale trial in which the intervention is delivered by people with substantially less expertise.(7, 8)

Intervention studies of health behaviors have common features that lend themselves to the introduction of RGBs. All interventions are delivered to a specified population (i.e., target audience) by a particular person (i.e., delivery agent) for a specified amount of time (i.e., intervention duration) with some form of support to implement. Such features, as tested in a preliminary study, are often altered in the larger trial. For example, interventions may be tested in high-income populations during a preliminary study but delivered to more socio-economically diverse populations in larger trials. These RGBs, as well as others, are expected to be associated with reduced intervention effectiveness in larger trials where the RGBs have been removed.(5, 6)

To date, information about the prevalence and impact of RGBs has come from the obesity literature.(5, 6) an important question is: Do RGBs exist and operate in intervention studies of other behaviors, as they do in the obesity literature? Interventions designed without RGBs produce more reliable effect estimates (e.g., experience smaller voltage drop)(5, 6) when tested on a larger-scale, and have a greater likelihood of leading to improved impact on population health. Identifying whether interventions targeting diverse health behaviors contain RGBs would provide evidence of the widespread use and impact of RGBs and could provide insights into potential causes for the introduction of such biases.

We aimed to establish the presence and impact of RGBs in interventions that aim to influence behaviors outside of those that are implicated in obesity (e.g., physical activity, nutrition). The areas of tobacco use disorder, alcohol use disorder, interpersonal violence, and sexually transmitted infections were chosen because their outcomes are distinct from those in obesity research, though interventions developed to address these topics follow the same intervention development patterns, with preliminary studies informing larger trials. (9–11) The differences in outcomes, but similarities in developmental processes allowed us to identify if RGBs are universal within behavior intervention development or a problem specific to the domain of obesity. We hypothesized RGBs would be present and adversely impacting other areas of health behavior intervention research. If our hypothesis is confirmed, we expect interventions containing RGBs would experience larger decreases in effectiveness (e.g., larger decrease in standardized mean difference [ΔSMD]) relative to interventions that do not contain RGBs.

## Method

We followed the same process we used in previous meta-epidemiological studies of RGBs in childhood obesity and adult obesity studies.(5, 12) Our methods were informed by the Cochrane Handbook for Systematic Review of Interventions(13) and are reported, where applicable, according to the Preferred Reporting Items for Systematic review and Meta-Analysis (PRISMA) Scoping Review and Abstract Extension statements (**Additional File 1**).(14, 15) Consistent with our prior work, a preliminary health behavior intervention represents an initial evaluation of a behavior-focused health intervention with primary goals to test the feasibility, acceptability, preliminary efficacy (or effect sizes) or other developmental features of the intervention.(5) Health behavior interventions were defined as coordinated sets of activities that aim to promote a health behavior by targeting one or more levels of influence, including interpersonal, intrapersonal, policy, community, macro-environments, micro-environments, and institutions.(16–19)

### Data Sources & Search Strategy

Our team used the following procedures to identify pairs of preliminary studies and their subsequent larger trial of the same or similar behavior intervention addressing tobacco use disorder, alcohol use disorder, interpersonal violence, or behaviors related to increased sexually transmitted infections. In Step 1, we used controlled vocabulary terms (e.g., MeSH and Emtree), free-text terms, and Boolean operators to identify systematic reviews and/or meta-analysis across OVID Medline/PubMed; Embase/Elsevier; EBSCOhost; and Web of Science databases.(20) The search strategy and syntax are provided in **Additional File 2**. In Step 2, our team uploaded identified systematic reviews and/or meta-analysis into an EndNote Library (v. X9.2) where they were reviewed by at least one trained research assistant (LV, KR) prior to retrieving full-text articles of all included studies within each review. In step 3, we retrieved the full-text articles included within each systematic review and/or meta-analysis uploaded them into NVivo (v.12, Doncaster, Australia). NVivo text search query was used to identify each study included within each systematic review and/or meta-analysis as either a (1) self-identified preliminary testing of an intervention (e.g., contained the words “pilot”, “feasibility,” “preliminary,” “proof-of-concept,” “vanguard,” “novel”, or “evidentiary” (16, 21, 22) or (2) a larger-scale trial referring to prior preliminary work to flag sections of text (e.g., “protocol” “previously”, “rationale”, “elsewhere described”, “prior work”, “informed by”). In Step 4, we used forward and backward citation searches to pair studies. Studies identified as large-scale trials were “followed back” using the references in the publication to identify preliminary testing and publication of an intervention within the body of the article. Studies identified as preliminary studies were “followed forward” using the Web of Science Reference Search interface (e.g., identify subsequent published studies referencing the identified preliminary study as informative preliminary work). Successfully paired preliminary studies and large-scale trials were catalogued in Excel (Microsoft) and referred to as ‘study pairs.

### Inclusion/Exclusion Criteria

Included pairs had to contain at least one self-identifying preliminary study and one larger-scale trial of the same or refined intervention. Studies had to be published in indexed, refereed journals as verified by Ulrich’s Web (http://ulrichsweb.serialssolutions.com). Studies had to be available as a full-text article in English. No participant age requirements or date boundaries were applied. To be included in our analysis, study pairs had to report the following: point estimates and measures of variance for the outcomes (e.g., SD, SE, 95%CI). Preliminary studies reporting only feasibility data (e.g., attendance, adherence, acceptability) could not be included in the quantitative analysis because they did not provide the necessary data to calculate an effect size. Additionally, study pairs had to present one shared outcome in both the preliminary study and the larger trial. For example, if a preliminary study reported intention to quit smoking and a larger trial reported quit rates, then these studies could not be used because the data reported in the larger trial could not be logically combined with the preliminary study’s data to produce consistent information about the phenomena of interest (e.g., the health behavior outcome - tobacco use). Where study pairs contained more than one eligible outcome, all outcomes were retained. Hierarchical models (see analysis) were used to account for lack of independence between outcomes from the same study pair.

### Study Outcomes

To procure summary statistics comparable across all studies, outcomes reporting impact on health-related behaviors were extracted by the research team (LV, KR, MS, SB, CDP). Tobacco cessation rates (i.e., quit rates) were extracted from tobacco use disorder studies (e.g., 7-day point prevalence, exhaled carbon monoxide levels, self-reported quit rate). Drinking rates were extracted from alcohol use disorder studies (e.g., ASI Alcohol Composite score, units of alcohol consumed over the previous week). Measures of interpersonal functioning were extracted from studies targeting interpersonal violence (e.g., nonviolent discipline, physical victimization, child abuse potential inventory score). Measures representative of constructs associated with reduced infection rates (e.g., transmission risk behaviors, medication adherence, psychosocial functioning) were extracted from studies on behaviors related to increased sexually transmitted infection.

### Coding Risk of Generalizability Biases

At least two reviewers (LV, MB, SB) independently reviewed each study pair to identify the presence/absence of RGBs using definitions presented in prior work (Table 1).(5, 12) Where discrepancies in coding RGBs occurred between reviewers, a third reviewer was consulted, and agreement was reached by discussion. Within each study pair, each RGB could be classified as not present, present in the preliminary study only, or present in preliminary study and larger trial (i.e., carried forward). Intervention duration, intervention intensity, and measurement bias are biases describing difference between preliminary study features and larger trials and, if present, were coded as present in both the preliminary study and larger trial. For studies where an RGB was present in the larger trial but not the preliminary study, for example, where implementation support was provided in the larger trial but was not mentioned in the preliminary study, it was assumed to have also been provided in the preliminary study, even if noy explicitly mentioned ad was coded in both the preliminary study and larger trial.

### Analytic Procedures

Consistent with previous studies,(5, 6) our research team extracted outcomes reported across pairs and entered them into an Excel file (Microsoft). In Excel, effect sizes were corrected for differences in the direction of the scales so that positive effect sizes corresponded to improvements in health behaviors in the intervention group. This was done for the simplicity of interpretive purposes so that all effect sizes could be summarized and compared within and across studies. We performed all necessary data transformations in Excel (e.g., standard errors and confidence intervals transformed into standard deviations). Next, outcomes reported within pairs were transferred into Comprehensive Meta-Analysis software (Biostat Inc., v3.3.07) to calculate the standardized mean difference (SMD) for each study. After effects were calculated, the complete data file was exported as a .CSV and uploaded into STATA 16 (SE, StataCorp) for analysis (LV, MB).

The natural hierarchical structure of the data is effects (Level 1) nested within studies (Level 2), which are nested within pairs (Level 3). However, three-level meta-regression models, to the best of our knowledge, have not yet been created and tested and we had to utilize two-level meta-regressions for all estimates. A random-effects meta-regression model with robust variance were used to compare the change in SMD (column labeled “ΔSDM” in **Additional File 3**). For these models, estimates of ΔSDM were nested within study pairs because, the change in effect size is a attribute of a study pair, not a single study. Random-effects meta-regression model with robust variance were also used to generate summative effect estimates for preliminary studies and larger trials (columns labeled “Preliminary Studies” and “Larger Trials” in **Additional File 3**), though effects were nested within a study because for the purpose of the model, an effect(s) is a property of a study independent of the existence of a study pair. These models were repeated for all levels of each RGB such that each row and column in **Additional File 3** represents a single model.

The difference in the SMD from the preliminary and larger scale trial were quantified according to previously defined formulas for the scale-up penalty.(4, 23, 24) This was calculated as: the SMD of the larger-scale trial divided by the SMD of the preliminary study and multiplied by 100. A value of 100% indicated identical SMDs in both the preliminary and larger-scale trial. A value of 50% indicated the larger-scale trial was half as effective as the preliminary study; a value above 100% indicated the larger-scale trial was more effective than the preliminary, whereas a negative value indicated the direction of the effect in the larger-scale trial was opposite of the preliminary. In line with prior work, a secondary evaluation of the impact of the biases was performed examining whether the presence/absence of biases was associated with nominally statistically significant outcomes (i.e., p ≤ 0.05) in the larger-scale trials.

## Results

### Descriptive Analysis

**Figure 1:**
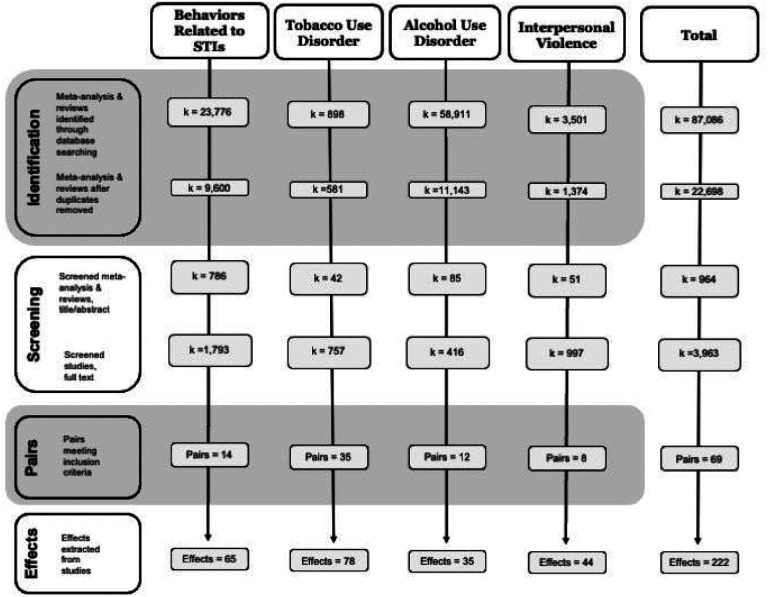
Modified PRISMA flow diagram of systematic literature search.

A modified PRISMA diagram detailing the search progression for each topic is presented in Figure 1. (14) Systematic searches identified 22,698 unique records, which resulted in 69 study pairs comprised of 138 studies, producing 222 effects (references provided in **Additional File 4**). For 44 pairs there were two outcome effects, for 8 pairs there were three or more effects, 17 pairs had four or more effects.

Across all 69 study pairs, 16% (n=11 pairs) were coded as containing no RGBs, 46% (n=32 pairs) contained at least one RGB and 36% (n=25 pairs) contained two or more RGBs. The most common biases were implementation support bias (38%, n=26 pairs), delivery agent bias (20%, n=14 pairs), followed by duration bias (13%, n=9 pairs), intensity bias (12%, n=8 pairs) and setting bias (12%, n=8 pairs). Audience bias was least common, being present in 7% (n=5) of study pairs. The prevalence of RGBs within each discipline was not materially different and it is displayed in **Additional File 4**.

### Meta-Regression

Of these 69 pairs, 12 study pairs (29 effects) were excluded from analyses because the studies did not report usable data in the preliminary study or larger trial (e.g., reported only feasibility data, did not report a measure of variability). Ten study pairs (25 effects) provided single-group, post-only data and were analyzed separately from the main analyses because they reported proportions and did not provide a measure of variance, therein precluding them from being combined with standardized effect sizes (**Additional File 5**).(25)

**Figure 2:**
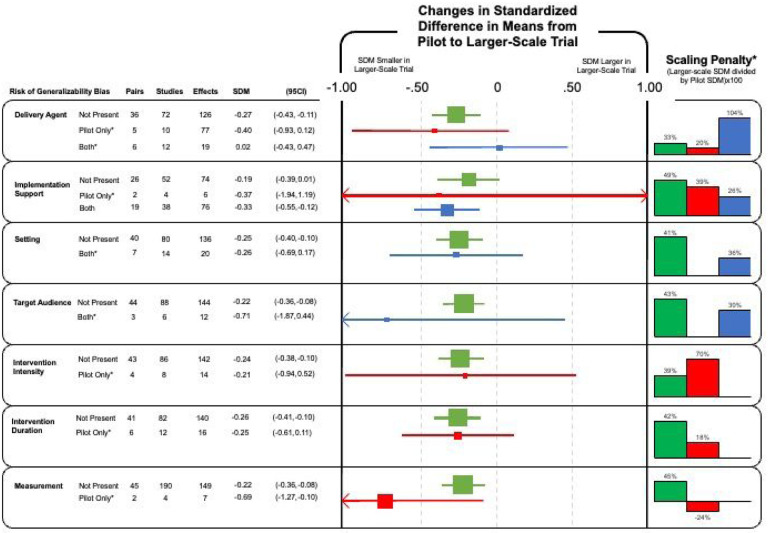
Change in the standardized difference in means of the presence (red), absence (green) or carried forward (blue) risk of generalizability from a preliminary study to a larger trial. Footnote: * Indicates estimates should be interpreted with caution due to degrees of freedom ≤ 4. (33)

In total, 47 study pairs producing 156 unique effects were eligible for inclusion in the meta-regression analyses and are represented in Figure 2. For study pairs where no RGBs were present, the effect size decreased by an average of ΔSMD=−0.24 (range −0.19 to −0.27). For pairs where the RGB was present in the preliminary study but removed in the larger trial (shown in red in Figure 2), the effect size decreased by an average of ΔSMD=−0.38 (range −0.69 to −0.21). For study pairs where RGBs were coded as present in the preliminary study *and* in the larger trial (i.e., carried forward; shown in blue in Figure 2), the effect size decreased by an average of ΔSMD =−0.19 (range −0.71 to 0.02). Details concerning specific model outputs can be found in **Additional File 3.**

The scale-up penalties associated with the RGBs are also presented in Figure 2 (far right). Interventions were generally less effective in larger trials compared to preliminary studies. This pattern was seen for 15/16 analyses, with 14 having smaller effects in large trials and in one even the direction of effect being reversed in the larger trials. For study pairs without bias, the scale-up penalties ranged from 33% to 49% (i.e., 51–67% relative reductions in the effect size in larger trials versus pilot studies). For study pairs where the RGB was carried forward the penalties ranged from 26% to 104%, and for studies containing bias in the preliminary study only it ranged from −24% to 70%. Overall, interventions were less effective at scale, except for delivery agent bias, where carrying forward the bias (i.e., the research team delivered the intervention in both the preliminary study and larger trial) corresponded to an increase in intervention effectiveness in the larger trial. There was no impact of RGBs on the odds of nominally statistically significant outcomes, defined as p ≤ 0.05 (**Additional File 6**).

## Discussion

RGBs are intervention features that are typically associated with diminished effectiveness in larger trials when they are present in preliminary studies but not in larger trials. RGBs operate within obesity interventions,(5, 12) but given the ubiquity of intervention features across different health behavior interventions, RGBs may operate in other fields beyond obesity. The purpose of this study was to establish whether RGBs were prevalent and impacting multiple disciplines of health behavior research.

Consistent with our hypothesis, RGBs were present and usually negatively impacted intervention effectiveness. Specifically, preliminary studies containing RGBs experienced larger decreases in effectiveness (i.e., ΔSMD) relative to interventions that do not contain RGBs although the difference was small. The presence of RGBs across multiple health behavior intervention areas indicates RGBs are not isolated to a single discipline but appear to be universal in their introduction. The ubiquity of RGBs indicates a shared aspects of intervention development, such as features of the research enterprise (e.g., timelines, budgets) and translational paradigms (e.g., models for testing interventions), may drive the introduction of RGBs in preliminary behavior interventions.

One such driver may be the need to produce compelling preliminary evidence to secure larger grant funding. Preliminary studies are considered critical to successfully competing for larger research grants. (26, 27) Scientific reviewers favor statistically significant findings,(28) rating them as more likely to produce meaningful results in subsequent fully powered studies, and having more justification for further testing.(29) Hence, it is plausible researchers, whether intentionally or unintentionally, introduce RGBs in their preliminary trials to maximize the odds of generating statistically significant results (e.g., p<0.05) to ensure a compelling grant application. (12, 30)

Research timelines may also contribute to the inclusion of RGBs in preliminary studies. Across disciplines, a myriad of factors work together to generate circumstances where project teams have limited opportunities to retest and refine an intervention before using it to support a grant application for a larger trial. For example, an early stage investigator may need to procure external funding within five years to secure tenure.(31) It may take 2–3 years to plan, conduct, and analyze a preliminary study with a further 2–3 years to apply and receive funding for a larger trial since successfully securing funding often takes multiple grant (re)submissions.(32) This timeline leaves little room for additional testing of an intervention that could allow for intervention refinement or replication as promoted by intervention development guidelines.(16)

Budget-driven constraints may also make it difficult to avoid introducing RGBs in preliminary studies. Funding for preliminary studies often comes from internal sources (e.g., a university) or career development award (e.g., National Institute of Health K awards) with limited direct research funds.(29, 31) Principal Investigators or graduate students may serve as the delivery agents during a preliminary study because of insufficient funds to hire staff to deliver the intervention, therein introducing delivery agent bias. Budgets can also dictate how participants are recruited and where an intervention is delivered (e.g., limited or no incentives, on university campuses), attracting participants that are systematically different than the eventual target population, introducing audience and setting bias. However, while tight budgets may seem like an obvious reason for the introduction of RGBs in preliminary studies, interventions for youth obesity indicate the prevalence of RGBs is not associated with preliminary study budgets. Preliminary studies with both small (e.g., doctoral student award) and large (e.g., National Institutes of Health [NIH] funded R21 or R01) budgets contain similar rates of RGBs,(5) indicating funding alone is not responsible for the introduction of bias.

Lastly, health interventions often follow translational frameworks like the NIH ORBIT Model,(16) the NIH Stage Model of Behavioral Intervention Development, (9) the NIH Common Fund Science of Behavior Change Experimental Medicine Approach,(10) and Greenwald and Cullen’s cancer control phase model developed for the National Cancer Institute.(11) Translational paradigms promote internal validity (i.e., efficacy) in earlier development stages, suggesting the testing of interventions under “optimal conditions”,(16) and place specific focus on identifying the mechanism(s) driving interventions.(10) In alignment with these frameworks, researchers may deliver preliminary studies under more tightly controlled conditions, with easier-to-reach populations (e.g., higher SES), and engage with participants more regularly to ensure intervention fidelity (i.e., implementation support). As studies transition to the later stages of translational frameworks, the emphasis shifts to external validity (i.e., generalizability). The latter stages focus on testing the intervention in the general population or “real world” setting. This may prompt a PI to deliver the intervention with less oversight (i.e., delivery agent and/or implementation support bias) to a more general population (i.e., target audience bias). The shift from internal validity to external validity promoted by common intervention development frameworks may inadvertently imbed the RGBs into intervention development, driving the inclusion of study features known to lead to diminished effectiveness.

### Limitations

Though all effects were in the expected direction (i.e., study pairs containing RGBs tended to have larger ΔSMD) the small number of studies (n=47) and hierarchical data structure contributed to wider 95% confidence intervals and studies containing RGBs did not have markedly different ΔSMD than those not containing RGBs. While this is consistent with the patterns observed in the obesity related interventions, (5, 12) it should be noted these estimates should be interpreted with caution due to the small number of pairs in each of the presented models.(33) Indeed, we did not find enough study pairs to meaningfully analyze RGBs and their effects in specific behavior areas (see **Additional File 4**), and these issues may be more prevalent in some areas than others. Nevertheless, the relative prevalence of study pairs in the present study were comparable to other similar meta-epidemiological studies,(34, 35) indicating our search process was successful at identifying eligible study pairs.

It should also be noted that incomplete study reporting may have limited the findings of this study. Articles routinely provide incomplete information about critical components of their interventions, such as who delivers an intervention, which may lead to the under identification of some biases.(36–39) It is not possible to distinguish between incomplete reporting and the absence of an RGB, therefore the present estimates of the prevalence and impact of RGBs may be conservative relative to their true prevalence rate. Finally, we should caution that pilot studies may be more susceptible to selective reporting biases. (30) Therefore, some of the voltage drop in the reported effect sizes in larger trials may reflect these biases operating more prominently in the pilot trials’ literature. Then, we cannot exclude that some RGBs may be more closely associated with selective reporting patterns, thus causing in part some of the observed associations.

## Conclusion

This cross-disciplinary analysis provides evidence of the presence and potential impact of the RGBs across multiple areas of health behavior interventions. The ubiquity of the RGBs indicates their presence may arise from underlying paradigms and practices common across behavior intervention fields, such as funding procedures and intervention development frameworks which facilitate or promote the introduction of RGBs in preliminary studies. Efforts to explore and address these system-level drivers of bias could lead to more consistent results between pilot and larger trials and help understand how to obtain and use evidence from trials to improve public health outcomes.

## Figures and Tables

**Table 1:** Operational definitions of the Risk of Generalizability biases.

Bias	Operationalized Definition
Delivery Agent Bias	Difference(s) in the level of expertise of the individual(s) who deliver the intervention in the preliminary study compared to who will deliver the intervention in larger trial(s).
Implementation Support Bias	Difference(s) in the amount of support provided to implement the intervention
Setting Bias	Difference(s) in the setting where the intervention is delivered in the preliminary study compared to who will deliver the intervention in larger trial(s).
Target Audience Bias	Difference(s) in the demographics of those that received the intervention in the preliminary study compared to who will deliver the intervention in larger trial(s).
Intervention Intensity Bias	Difference(s) in the number and length of contacts in the preliminary study compared to who will deliver the intervention in larger trial(s).
Intervention Duration Bias	Difference(s) in the length of the intervention provided in the preliminary study compared to who will deliver the intervention in larger trial(s).
Measurement Bias	Difference(s) in the measures employed in the current study and the measures used in the preliminary study compared to who will deliver the intervention in larger trial(s).

Note: Table 1 based on definitions originally appearing in Beets et al. 2020(5)

## Data Availability

Available upon reasonable request to the corresponding author.

## Abbreviations

RGBs: Risk of Generalizability Biases
SMD: Standardized Mean Difference
NIH: National Institutes of Health
ORBIT: Obesity-Related Behavioral Intervention Trials model
